# Uncovering New Mutations Conferring Azole Resistance in the *Aspergillus fumigatus cyp51A* Gene

**DOI:** 10.3389/fmicb.2019.03127

**Published:** 2020-01-21

**Authors:** Peiying Chen, Musang Liu, Qiuqiong Zeng, Zheng Zhang, Weida Liu, Hong Sang, Ling Lu

**Affiliations:** ^1^Department of Dermatology, Jinling Hospital, School of Medicine, Nanjing University, Nanjing, China; ^2^Department of Medical Mycology, Institute of Dermatology, Chinese Academy of Medical Sciences and Peking Union Medical College, Nanjing, China; ^3^Jiangsu Key Laboratory of Molecular Biology for Skin Diseases and STIs, Nanjing, China; ^4^Jiangsu Key Laboratory for Microbes and Functional Genomics, Jiangsu Engineering and Technology Research Center for Microbiology, College of Life Sciences, Nanjing Normal University, Nanjing, China

**Keywords:** *Aspergillus fumigatus*, azoles, drug resistance, mutations, Cyp51A

## Abstract

The opportunistic pathogen *Aspergillus fumigatus* has developed worldwide resistance to azoles largely through mutations in cytochromeP450 enzyme Cyp51. In this study, we indicated that *in vitro* azole situation results in emergence of azole-resistant mutations. There are previously identified azole-resistant *cyp51A* mutations (M220K, M220I, M220R, G54E and G54W mutations) and we successfully identified in this study two new mutations (N248K/V436A, Y433N substitution) conferring azole resistance among 18 independent stable azole-resistant isolates. The *Galleria mellonella* model of *A. fumigatus* infection experiment verified that Cyp51A mutations N248K/V436A and Y433N reduce efficacy of azole therapy. In addition, a predicted Cyp51A 3D structural model suggested that Y433N mutation causes the reduced affinities between drug target Cyp51A and azole antifungals. This study suggests that drug selection pressure make it possible to isolate unidentified *cyp51A* mutations conferring azole resistance in *A. fumigatus*.

## Introduction

*Aspergillus fumigatus*, the most common *Aspergillus* species, is a major opportunistic fungal pathogen and may lead to a variety of allergic reactions and life-threatening systemic infections in humans, including invasive pulmonary aspergillosis, pulmonary aspergilloma, and allergic bronchopulmonary aspergillosis (Hagiwara et al., 2016; Chowdhary et al., 2017). Antifungal drugs are limited for treatment options and they include three classes: polyenes (amphotericin B), azoles and echinocandins. To date, azoles are the preferred agents for first-line prophylaxis and treatment of aspergillosis (Walsh et al., 2008; Garcia-Rubio et al., 2017). The treatment of invasive infections has proved to be difficult because of limited antifungal drugs and occurrence of antifungal-resistant strains worldwide and the host immunocompromised status (Shao et al., 2007). It is reported that clinical long-term azole therapy tends to promote the emergence of the azole-resistant strains (Camps et al., 2012; Tashiro et al., 2012; Chowdhary et al., 2017). However, for the treatment and effective maintenance therapy of chronic aspergillosis, prolonged antifungal therapy is needed (Godet et al., 2016). Thus, further understanding of the molecular mechanism of azole resistance in *A. fumigatus* in an azole-exposure environment is of great clinical importance and biological interest.

Previous studies indicated that point mutations of *cyp51A* coding for cytochrome P450 14α-sterol demethylase are the predominant mechanisms of resistance to azole drugs in *A. fumigatus* (Becher and Wirsel, 2012; Vazquez and Manavathu, 2016; Chowdhary et al., 2017). To date, except for the hot spots G54, G138, M220, and G448, more than 20 different amino acid substitutions (single or multiple amino acid change in the same strain) in the Cyp51A protein have been found in clinic-isolated and laboratory-isolated azole-resistant strains (Bowyer et al., 2011; Arendrup, 2014; Cowen et al., 2015; Sharma et al., 2015). Genetic reconstitution experiments have verified that the mutations G54A, G54W, P216L, M220V/K/T, and G448S are related to azole resistance (Mann et al., 2003; Camps et al., 2012; Krishnan Natesan et al., 2012). In addition, it has been reported that many unexplored non-*cyp51A* mutations are related to *A. fumigatus* resistance (Chowdhary et al., 2014; Moye-Rowley, 2015; Wei et al., 2017). In clinical cases, among serially isolated several strains from one patient receiving azole treatment, the firstly isolated strains exhibited azole susceptibility without any *cyp51A* mutations, however, subsequent isolates showed azole resistance associated with *cyp51A* mutations (Chen et al., 2005; Howard et al., 2009; Camps et al., 2012; Tashiro et al., 2012). *A. fumigatus* have remarkable ability to grow in diverse environments. Strong adaptability of fungi to the environment facilitates persistence of the species and compromises treatment option, even therapy failure, in the clinic (Verweij et al., 2016). Indeed, our screening experiment in this study further indicated that even though the high-dose azole are used *in vitro*, there are still a small number of azole-resistant mutants surviving in azole situation and there are unexplored *cyp51A* mutations conferring azole resistance in *A. fumigatus*.

## Materials and Methods

### Strains, Media, and Culture Conditions

The clinical *A. fumigatus* isolates NO.3 and NO.7 were previously isolated from two different immunocompetent patients with postoperative lung infections. The patients were not treated with azole until the strains were isolated and there is no drug environmental stress due to azole pesticides. The isolates were identified strictly by molecular sequencing of the ITS, β-tubulin, and calmodulin gene, and by the growth phenotype. The GenBank accession numbers for sequences of ITS, β-tubulin, and calmodulin of clinical isolates NO.3 is respectively MH536092, MH536090, and MH536094. Those for NO.7 is respectively MH536093, MH536091, and MH536095. The clinical isolates (NO.3 and NO.7) and mutant isolates are stored in CMCC (China Center for Medical Microorganisms Culture Collection, Institute of Dermatology, Chinese Academy of Medical Sciences and Peking Union Medical College, Nanjing, China). *A. fumigatus* A1160 strain (*ku80 pyrG*) was purchased from Fungal Genetics Stock Center. The strain A1160^C^ (*ku80* A1160:pyrG) belonged to a laboratory strain generated from reference strain A1160 transformed with *pyrG* selectable marker (Jiang et al., 2014). All *A. fumigatus* strains used in this study were grown on the YAG or YUU medium according to the growth needs of strains. YAG: 5 g/L Yeast extract, 2% glucose, 1 ml/L Trace elements, 2% agar. YUU medium is YAG medium plus uracil (1.1 g/L) and uridine (1.2 g/L). RPMI 1640 medium is composed of 10.4 g/l RPMI 1640 (catalog no. 31800-014; Invitrogen Gibco) and 34.53 g/l MOPS [3-(*N*-morpholino) propanesulfonic acid]. All strains were cultured at 37 or 35°C (according to the M38-A2 guidelines) for the times indicated. All the strains used in this study are listed in Supplementary Table S2 in the Supplementary Material.

### *In vitro* Screening of Itraconazole-Resistant Strains

First, parental strain NO.3 or NO.7 or A1160^C^ strains was cultured onto YAG medium for 3 days at 37°C. Then 5 million freshly harvested spores from one parental strain were spread onto medium (5 × 10^6^ spores/plate; 50 plates) supplemented with 4 μg/ml itraconazole and cultured them for 5 days. The surviving isolates on plates were serially streaked and cultured on itraconazole-containing plates (16 μg/ml) continuously four generations with an incubation period of 5 days at 37°C. The surviving strains on the final-step plate amended with 16 μg/ml itraconazole were cultured on drug-free medium continuously ten generations and then were spotted back onto medium with 16 μg/ml itraconazole. Isolates with stable drug resistance finally were named and kept for further studies.

### Antifungal Susceptibility Testing

All of the antifungal drugs were purchased from Sigma. Testing for susceptibility to the drug was carried out by the broth-based microdilution methods according to the CLSI document M38-A2 guidelines (Clinical and Laboratory Standards Institute, 2008). Briefly, conidial suspensions were diluted in RPMI1640 liquid medium and then transferred into a 96-well plate containing drug dilutions. The plates were incubated at 35°C for 48 h. The MIC was defined visually and by microscopy as the lowest concentration that prevented any discernible growth. *A. fumigatus* ATCC MYA-3626 and AF293 were included in related tests as quality control and reference strains. For the test of antifungal susceptibility in the constructed point mutant strains PY01 and PY02, RPMI1640 minus uracil and uridine liquid medium was used in order to maintain the growth of control stain A1160.

### The *cyp51A* Gene Sequence Analysis

The *cyp51A* gene coding region and its promoter were amplified by PCR with two pairs of primers cyp51A-F1/cyp51A-R1 and cyp51A-F2/cyp51A-R2. Gene c*yp51A* nucleotide sequence (AFUB_063960) of *A. fumigatus* 1163 reference strains from Ensembl Fungi database^1^ was used as reference sequence. Primers used in this study are listed in Supplementary Table S1.

### Cyp51A 3D Structural Model Analysis

The Cyp51A 3D structural model used in this study was constructed as described previously (Liu et al., 2016). Briefly, in order to investigate the structural perturbations caused by each mutation of Cyp51A, we performed single residue mutations respectively. For each mutation, we constructed the mutated structure and performed subsequent energy minimization based on the previously minimized wild type structure and computed the structural instability. The energy minimization was carried out with an Amber12: EHT force field that allows atoms in the mutated residue and neighboring residues within 10 Å to be free to move and other atoms fixed.

### Construction of *A. fumigatus cyp51A^*N*248*K/V*436*A*^* and *cyp51A^*Y*433*N*^* Mutant Strains

In an attempt to assess whether ITC resistance in *A. fumigatus* was the result of the mutations of Cyp51A N248K/V436A or Y433N, the strains containing *cyp51A* gene mutation sites were created. To construct strain PY01 (*cyp51A^*N*248*K/V*436*A*^*), the 5′-flanking region and ORF of *cyp51A^*N*248*K/V*436*A*^* from isolated strain NO 3-339 were amplified with primers cyp51A-F1 and P3. The 3′-flanking region of the *cyp51A^*N*248*K/V*436*A*^* gene was amplified from strain NO 3-339 with primer pair P4/P6, and the *Neurospora crassa* pyr4 gene (a selectable marker) was amplified from plasmid pLA5 with primer pair Pyr4-up/Pyr4-down. The three PCR products were fused with the nested primer pair P2/P5 and then transformed into recipient strain A1160 (a standard *A. fumigatus* strain susceptible to azoles) to generate mutant strain PY01 (*cyp51A^*N*248*K/V*436*A*^*). Using the same method, to construct PY02 (*cyp51A^*Y*433*N*^*) stain, the 5′-flanking region and ORF of *cyp51A^*Y*433*N*^* from isolated strain NO 1160-73 were amplified with primers cyp51A-F1 and P3. The 3′-flanking region of the *cyp51A^*Y*433*N*^* gene was amplified from strain NO 1160-73 with primer pair P4/P6, and the *Neurospora crassa* pyr4 gene was amplified from plasmid pLA5 with primer pair Pyr4-up/Pyr4-down. The three PCR products were fused with the nested primer pair P2/P5 and then transformed into recipient strain A1160 to generate mutant strain PY02 (*cyp51A^*Y*433*N*^*). The transformants were confirmed by diagnostic PCR with corresponding primers pairs (see Supplementary Figure S1). The *cyp51A* gene in the corresponding transformants was further analyzed by PCR sequencing and alignment to confirm the success of gene replacement and point mutation. Primers used in this study are listed in Supplementary Table S1.

### Virulence and the Azole Susceptibility Assays of *A. fumigatus cyp51A^*N*248*K/V*436*A*^* and *cyp51A^*Y*433*N*^* Mutant Strains

*Galleria mellonella* (400 ± 50 mg body weight) were used and 15 caterpillars per group were employed. Larvae were stored in the dark at 37°C prior to use. The conidial suspensions were collected in water. Preliminary experiments were performed to define an appropriate range of inoculum concentrations that causes > 80% mortality after 72–96 h post-infection (data not shown). A hamilton syringe (Fisher Scientific, Madrid, Spain) was used to infect the larvae with 10 μl of the inoculum suspension (8 × 10^7^/ml) into the hemocoel of each *G. mellonella* via the last left proleg. Within 2 h of infection, 10 μl of antifungal solution was injected in a different proleg using the same technique. Following antifungal treatment, caterpillars were maintained in plastic containers at 37°C and larval survival was monitored daily. The larvae were considered dead when they did not respond to touch. Infected larvae injected with water were used as an untreated control group. Other control groups were also included: untouched and injected with PBS. Each experiment was performed at least three times and the results are reported as mean values. In order to simplify some of the figures, untouched control groups may have been omitted from the graphs. Stock solutions of antifungals were first prepared in dimethyl sulfoxide (DMSO) and dilutions were then made to obtain the selected concentrations in water for injection. Therapeutic dose of VRC (40 mg/kg), POS (10 mg/kg), ITC (25 mg/kg) were selected. The log-rank (Mantel-Cox) test was performed for survival analysis. A *p*-value < 0.05 was considered significant.

## Results

### A Small Number of Azole-Resistant Isolates Are Obtained From *A. fumigatus* Azole-Susceptible Isolates Under Drug Situation

**FIGURE 1:**
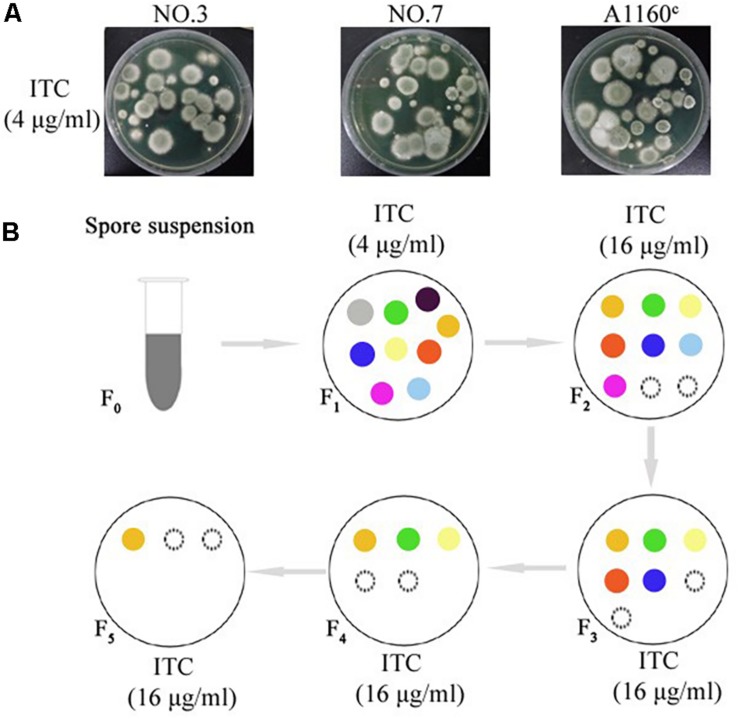
Induction of the adaptive strain. **(A)** 5 × 10^6^ freshly harvested spores (5 × 10^6^ spores/plate) from each parent strain were inoculated respectively on plates with 4 μg/ml itraconazole (ITC) at concentration that exert selection pressure for resistance to the drug at 37°C for 5 days. Different colony phenotype are shown on each plate. **(B)** The model of screening processes of the ITC-resistance strains. Extant lineages (F_1_ generation) under 4 μg/ml ITC were transferred into plates with 16 μg/ml ITC and incubated at 37°C for 5 days. Then, extant strains (F_2_ generation) on the 16 μg/ml ITC were continually transferred into next plates with 16 μg/ml ITC and incubated at 37°C for 5 days. Then, extant strains (F_3_ generation) were continually transferred into next plates with 16 μg/ml ITC, and so on. Extant lineages (F_5_ generation) on the plates with 16 μg/ml ITC were collected for further study. The colored circles represent different colonies. The black dotted line circles represent strains that show no signs of growth.

A screening experiment for azole resistance mutants was initiated using three azole-susceptible *A. fumigatus* isolates (named NO.3; NO.7 and A1160^C^) as the parent strains. Strains NO.3 and NO.7 were isolated from the patient sputum, and the strain A1160^C^ belonged to the laboratory strain generated from the clinical-derived reference strain A1160 (Jiang et al., 2014). As shown in Figure 1A, for each parent strain, 5 × 10^6^ spores/plate were inoculated on fifty plates with 4 μg/ml itraconazole and cultured for 5 days. The progenies of each parent strain showed different colonies with distinct phenotypes in 5 days after inoculation on 4 μg/ml itraconazole plates. Further, we attempted to investigate whether the growth of these drug-resistance isolates could be inhibited under high drug concentration. Therefore, *in vitro*, the extant progenies were propagated to first round of media with a high concentration of ITC (16 μg/ml) for a 5-day period, and then growing viable progenies were re-transfer to next round of 16 μg/ml ITC media for a 5-day period, and so on (Figure 1B). Most lineages failed to grow in the serial high-dose ITC environment. Eventually, there were still a small number of strains (18 isolates) surviving with stable drug resistance. They were isolated and kept for further studies.

**FIGURE 2:**
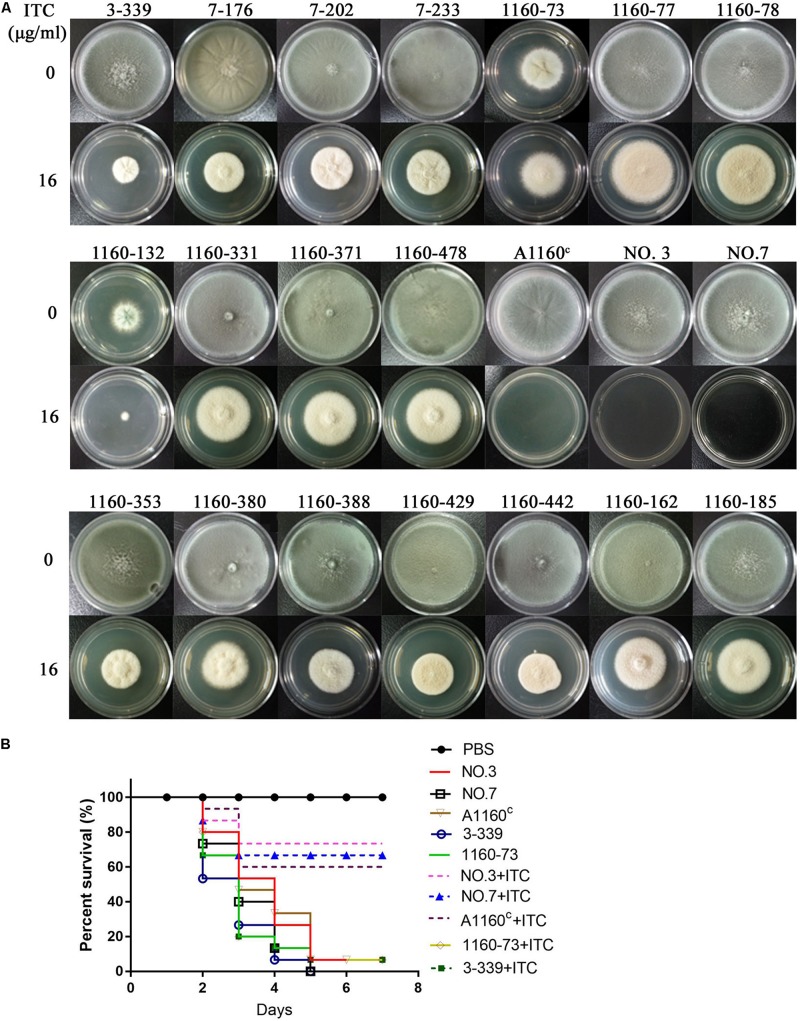
Colony phenotypes and azole susceptibilities of ITC-resistance isolates *in vitro* and *in vivo*. **(A)** Colony phenotypes of the 18 ITC-resistance isolates and the control strains on both drug-free media and media with high drug concentration (16 μg/ml ITC) at 37°C for 3 days. **(B)** Virulence and azole susceptibilities of isolates 3-339 (*cyp51A^*N*248*K/V*436*A*^*) and 1160-73 (*cyp51A^*Y*433*N*^*) and parent strains (NO.3, NO.7, A1160^C^) in a *Galleria mellonella* model.

Their colony phenotypes on both drug-free and drug-containing (16 μg/ml ITC) media were further observed. As shown in Figure 2A, two isolates (strain 1160-73, strain 1160-132) displayed reduced colony diameters while all other selected isolates showed almost similar levels of the colony diameter but with the different colony appearance compared to the reference strain AF293, suggesting that these isolates possibly belong to independent genotypes. Further, their resistance levels to other azoles (VRC, POS) were evaluated. As shown in Table 1, their MICs of ITC is 8-16 μg/ml and are higher than the ECV (epidemiological cutoff value, 1 μg/ml for itraconazole), suggesting their high-level resistant to ITC. And it can be calculated that emergence frequency of ITC resistance under long-term, high-dose azole stimuli was 0.4-5.6 × 10^–8^ (Table 2) in this *in vitro* experiment. In addition, all of these strains showed high-level POS resistance (MIC ≥ 1-16 μg/ml), and two strains were resistant to VRC (MIC = 4 μg/ml), suggesting usage of high-dose ITC may lead to cross-resistance phenomenon.

**TABLE 1:** MICs for triazoles and corresponding mutation type for *cyp51A.*

**Genotype of *cyp51A***			**MIC (μg/ml)**			
**Nucleotide Substitutions**	**Amino acid Substitutions**	**Strain**	**ITC**	**VRC**	**POS**	**Specimen type**
	**Control strains**						
		MYA3626	0.5	0.5	0.125	
		AF293	0.25	1	0.25	
	**Parent strains**						
T815A	N248K	NO.3	0.5	0.5	0.25	sputum
NM	NM	NO.7	0.5	0.5	0.25	sputum
NM	NM	A1160^C^	1	0.5	0.25	
	**Resistant mutants^∗^**						
T815A/T1378C	N248K/V436A	3-339	16	2	1	
T1368A	Y433N	1160-73	>16	4	8	
T730G	M220R	7-176	>16	1	2	
T730G	M220R	7-202	>16	1	2	
T730G	M220R	7-233	>16	1	2	
TG730AA	M220K	1160-77	>16	1	16	
TG730AA	M220K	1160-78	>16	1	16	
G731A	M220I	1160-162	>16	1	1	
G731A	M220I	1160-353	>16	1	1	
G731A	M220I	1160-380	>16	1	1	
G731A	M220I	1160-388	>16	1	1	
G731A	M220I	1160-429	>16	1	1	
G731A	M220I	1160-442	>16	1	1	
G161A	G54E	1160-185	>16	0.25	2	
G160T	G54W	1160-331	>16	0.25	>16	
G160T	G54W	1160-371	>16	0.25	>16	
G160T	G54W	1160-478	>16	0.25	>16	
NM	NM	1160-132	8	4	2	

*^∗^Resistant mutants obtained by drug exposure screening in this study. NM, no mutation.*

**TABLE 2:** Experimental percentage of resistance to azole yielded 18 ITC-resistant strains.

**Parent strain**	**Number of F_0_**	**Number of ITC resistance strains**	**Percentage of ITC resistance strains**
NO.3	5 × 10^6^ spores/plate × 50 plates	1	0.4 × 10^–8^
NO.7	5 × 10^6^ spores/plate × 50 plates	3	1.2 × 10^–8^
A1160^c^	5 × 10^6^ spores/plate × 50 plates	14	5.6 × 10^–8^

### New Amino Acid Mutations N248K/V436A and Y433N of the *A. fumigatus* Cyp51A Protein in Azole-Resistant Isolates Are Detected

Because *cyp51A* mutations is common reason for emergence of *A. fumigatus* azole resistance, we amplified entire *cyp51A* gene including its promoter region and sequenced using the primers (see Supplementary Table S1) for the above resistance isolates. Gene sequencing analysis showed that 17 isolates had mutations in *cyp51A* but only one strain retained the wild-type *cyp51A* gene, suggesting that the *cyp51A* mutation is still main mechanism of azole resistance and meanwhile non-*cyp51A*-mediated azole resistance existed. Gene sequencing data showed that these azole resistance isolates harbored seven types of Cyp51A mutations (N248K/V436A, Y433N, M220K, M220I, M220R, G54E and G54W). It is reported that M220K, M220I, M220R, G54E and G54W confer azole resistance and in our study these mutants displayed indeed high MIC value for ITC and POS (Table 1). Notably, the V436A (T1378C) and Y433N (T1368A) amino acid alterations have not been described previously in *A. fumigatus* azole-resistant isolates. We found that *in vitro* the isolate (3-339) carrying the N248K/V436A substitution shows resistant to ITC and POS but VRC-intermediate resistance and the isolate (1160-73) harboring Y433N mutation displays cross resistance to ITC, VRC and POS (Table 1). Additionally, as shown in Figure 2B, in fungal-infection *Galleria mellonella* model, ITC treatment fail to increase the survival of the larvae infected with the mutation 3-339 or 1160-73 (*p* > 0.05 between groups without and with ITC), compared with the group infected with the control parental strain showing an improvement in survival (*p* < 0.05 between groups infected with the parental strains without and with ITC).

### A Predicted 3D Structural Model Displays a Possible Resistance Mechanism for the New and Unexplored Cyp51A Mutation Y433N

**FIGURE 3:**
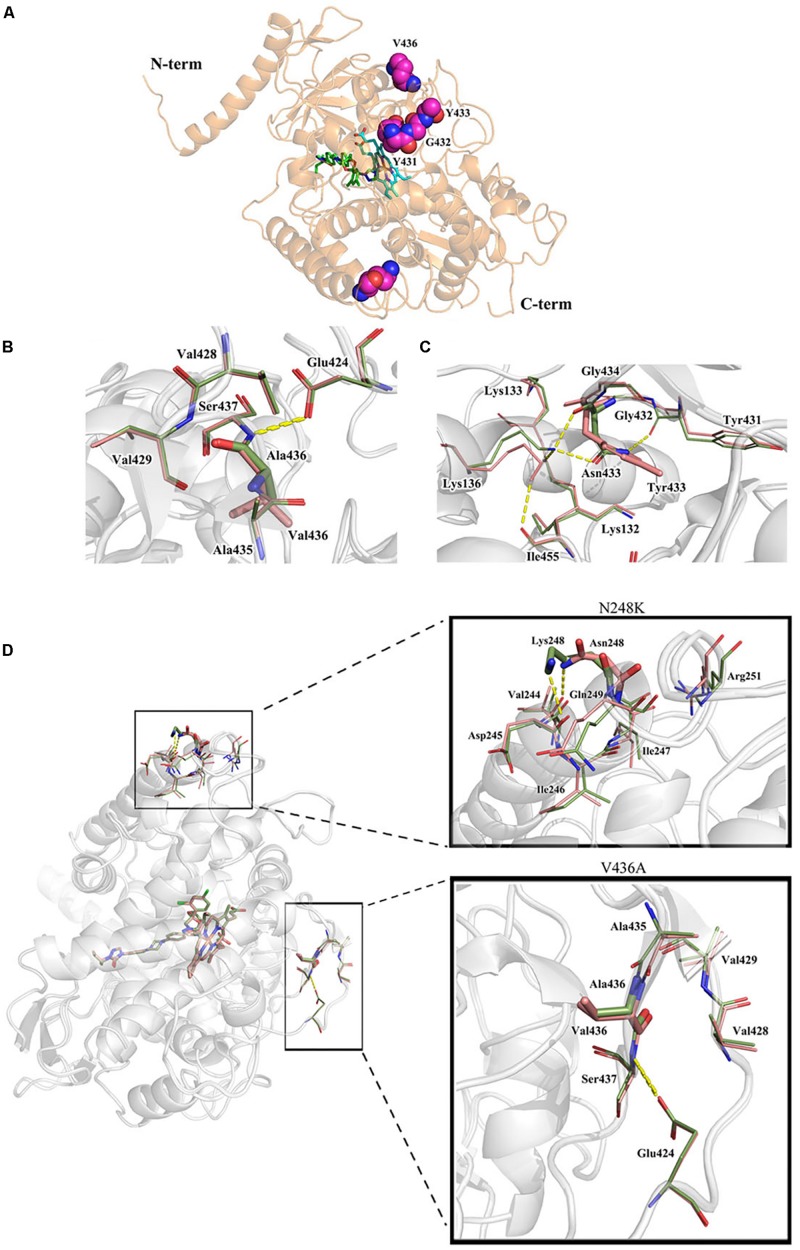
Overall homology modeling structure of *A. fumigatus* Cyp51A **(A)** and comparison of wild-type and mutant structures for Cyp51A of V436A **(B)** and Y433N **(C)** and N248K/V436A **(D)**. **(A)** The heme is shown as cyan sticks. Itraconazole is shown above the heme plane as green sticks. V436, Y433, Y431 and G432 (neighboring residues) are shown as magenta space-filling forms. **(B,C)** The wild-type structures are colored in salmon and the mutant are in smudge. Yellow dashed lines show the hydrogen bond interactions of amino acids. The V436A mutation brought about limited reduction of steric volume and hydrophobicity which had little influence on nearby residues and thus should not be associated with resistance. On the contrary, the mutation of Y433N significantly interfered nearby residues involving heme. Lys136 changed its orientation and reformed a hydrogen bond with Asn433 instead of Ile455 while the less bulky Asn433 allowed Lys132 forming a hydrogen bond with Gly450 which weakened the ionic interaction between Lys132 and one of the heme propionates. **(D)** Mutation N248K would change the orientation of the residue by reforming hydrogen-bond interactions. The wild-typed hydrogen bond formed by Asn248 with Val244 was lost in the mutant where one hydrogen bond between Asn248 and Asp245 was formed. The mutation V436A (change from Val436 to Ala436) slightly reduced the steric volume, leading to minor influence to nearby residues. The two mutations caused a change in the overall structure of Cyp51A, and affecting the conformation of the protein pocket, but the position of N248K and V436A is far from heme.

Both the strain NO.3 (parent strain) and mutant strain 3-339 harbor amino acid mutation N248K in Cyp51A (Table 1). In fact, N248K had been found in many azole-susceptible *A. fumigatus* strains, and it is believed that this site does not correlate with drug resistance (Liu et al., 2015). Therefore, we next focused on whether V436A and Y433N mutations analyzed by gene sequencing confer resistance to azole. In previous studies, the *A. fumigatus* Cyp51A protein homology modeling approach has been utilized to investigate the correlation between the drug binding site in Cyp51A mutation and azole resistance. A new protein structural model has been built based on a recently resolved crystal structure of the Cyp51A protein of *S. cerevisiae* that currently displays the highest homology (up to 50.7%) with the *A. fumigatus* Cyp51A protein (Liu et al., 2016). In the overall homology 3D modeling structure of *A. fumigatus* Cyp51A (Figure 3A), a typical P450 fold with a well-conserved structural core around the heme was shown. To investigate the structural perturbations caused by each Cyp51A mutation found in this study, we performed single residue mutation in the 3D structural model of the *A. fumigatus* Cyp51A protein (as shown in Figures 3B,C).

Comparison of wild-type and mutant structures for the Cyp51A of V436A is shown in Figure 3B. The change from Val436 to Ala436 slightly reduced the steric volume but had little influence on nearby residues, and both residues are hydrophobic. These results suggest that it would be difficult to deduce azole resistance with the mutation. Thus, whether the V436A mutation in the Cyp51A protein could confer azole resistance may need to be explored by genetic reconstitution experiments.

A comparison of the wild-type and mutant structures for Cyp51A of Y433N is shown in Figure 3C. Y433N would change the orientation of the residues by reforming hydrogen-bond interactions. A hydrogen bond of Asn433 with Lys136 was formed leading to the loss of hydrogen bonding of Lys136 with Ile455. The mutation also affected the hydrogen bonding network within nearby residues including Tyr121, Lys132, Gly450, and Arg451. The less bulky residue Asn433 allowed hydrogen bonding of Lys132 and Gly450 and thus weakened the ionic interaction between Lys132 and one of the heme propionates and the hydrogen bond between Tyr121 and the propionate. The loop right above the heme was thus affected, which influenced the affinities of antifungals so as to produce drug resistance. Therefore, based on this predicted 3D structural model, the Y433N substitution of Cyp51A maybe is related to azole resistance by reducing the affinities of the antifungals.

The strain 3-339 contains both mutations V436A and N248K. Therefore, the mutant structures for Cyp51A of V436A in combination with N248K was analyzed in protein modeling (Figure 3D). Mutation N248K would change the orientation of the residue by reforming hydrogen-bond interactions. The wild-typed hydrogen bond formed by Asn248 with Val244 was lost in the mutant where one hydrogen bond between Asn248 and Asp245 was formed. The mutation V436A (change from Val436 to Ala436) slightly reduced the steric volume, leading to minor influence to nearby residues. The two mutations caused a change in the overall structure of Cyp51A, and affecting the conformation of the protein pocket, but the details of the action are unknown because the position of N248K and V436A is far from heme. Thus, whether the N248K/V436A mutation in the Cyp51A protein could confer azole resistance may need to be explored by genetic reconstitution experiments.

### Point Mutations T815A/T1378C (N248K/V436A) and T1368A (Y433N) in the *cyp51A* Gene Confer Azole Resistance in *A. fumigatus* Using Gene Replacement Method

**FIGURE 4:**
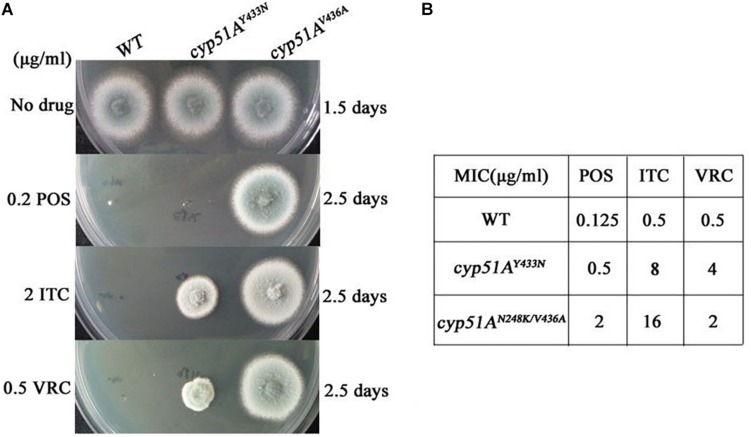
Point mutations *cyp51A^*N*248*K/V*436*A*^* and *cyp51A^*Y*433*N*^* confer azole resistance in *A. fumigatus*. **(A)** Susceptibilities of A1160 (WT), PY01 (*cyp51A^*N*248*K/V*436*A*^*) and PY02 (*cyp51A^*Y*433*N*^*) to azoles determined. 2 μl from 10^6^ spore suspension of these strains were spotted onto YUU medium containing antifungals. **(B)** MIC of POS, ITC and VOR for strains A1160 (WT), PY01 (*cyp51A^*N*248*K/V*436*A*^*) and PY02 (*cyp51A^*Y*433*N*^*).

Genetic reconstitution experiments were further performed to verify whether the T815A/T1378C (N248K/V436A) and T1368A (Y433N) contribute the azole resistance in *A. fumigatus*. Using a PEG-mediated gene transformation system of protoplasts, the *cyp51A* gene in strain A1160 (a standard wild-type *A. fumigatus* strain susceptible to azoles) was replaced with the one from mutant strain 3-339 (*cyp51A^*N*248*K/V*436*A*^*). Similarly, the *cyp51A* gene in strain A1160 was replaced with the one from mutant strain 1160-73 (*cyp51A^*Y*433*N*^*). The diagnostic PCR and sequencing analysis for *cyp51A* gene in the transformants showed that the mutated *cyp51A* gene (*cyp51A^*N*248*K/V*436*A*^* or *cyp51A^*Y*433*N*^*) had been integrated into at the original *cyp51A* gene locus in the genome of strain A1160 (Supplementary Figures S1, S2). As expected, our reconstructed strains PY01 (*cyp51A^*N*248*K/V*436*A*^*) and PY02 (*cyp51A^*Y*433*N*^*) showed obviously reduced susceptibility to azoles (Figure 4). Compared to that of the wild-type strain A1160, strain PY01 had a 32-fold increase in ITC MIC, 4-fold increase in VRC MIC, and 16-fold increase in POS MIC. Strain PY02 had a 16-fold increase in ITC MIC, 8-fold increase in VRC MIC, and 4-fold increase in POS MIC. These data collectively indicate that either *cyp51A^*N*248*K/V*436*A*^* or *cyp51A^*Y*433*N*^* was able to induce azole resistance. In addition, *cyp51A^*N*248*K/V*436*A*^* displayed phenotypes of resistance to POS and VRC-intermediate resistance, and *cyp51A^*Y*433*N*^* displayed phenotypes of resistance to VRC and POS-intermediate resistance, suggesting that *cyp51A^*N*248*K/V*436*A*^* and *cyp51A^*Y*433*N*^* may have different mechanisms of resistance to azole drugs in *A. fumigatus*.

### Virulence and Azole Susceptibilities of *cyp51A^*N*248*K/V*436*A*^* and *cyp51A^*Y*433*N*^* Mutants in a *Galleria mellonella* Model

**FIGURE 5:**
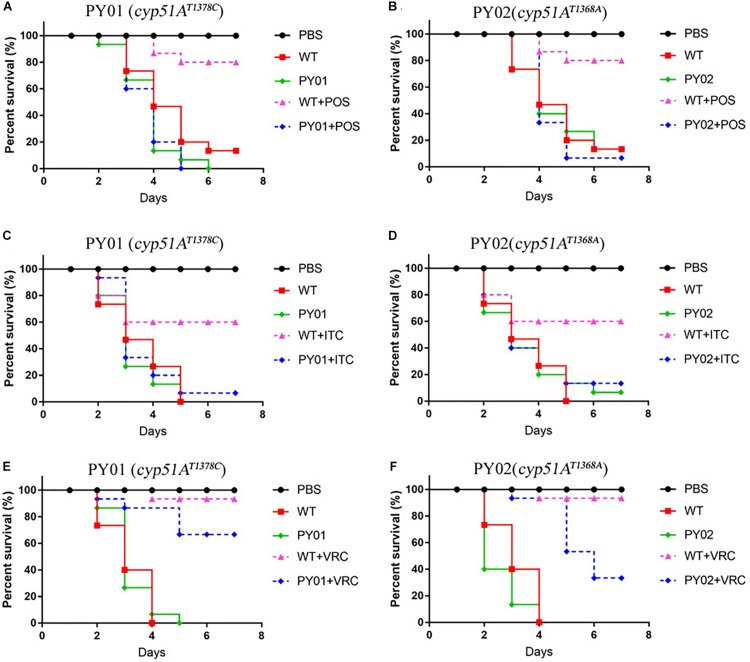
Virulence and azole susceptibilities of *cyp51A^*N*248*K/V*436*A*^* and *cyp51A^*Y*433*N*^* mutants in a *Galleria mellonella* model. **(A–F)** The survival rate curve of the *Galleria mellonella* infected by the mutants PY01 (*cyp51A^*N*248*K/V*436*A*^*) and PY02 (*cyp51A^*Y*433*N*^*) and the control parental wild-type strain with or without POS **(A,B)** or ITC **(C,D)** or VRC **(E,F)** administration.

To evaluate any effects of *cyp51A^*N*248*K/V*436*A*^* and *cyp51A^*Y*433*N*^* in an *in vivo Galleria mellonella* model, we examined virulence and the efficacy of azole therapy for the two reconstituted mutant strains PY01 and PY02. The group infected by the mutant PY01 or PY02 showed a mortality rate similar to the group infected with control parental wild-type strain (Figure 5), suggesting that there were no difference in virulence between them. Next, we verified whether strains PY01 and PY02 conferred resistance to azoles in *Galleria mellonella* model of *A. fumigatus* infection. Azoles (POS or ITC or VRC) was given via injection administration 2 h after fungal infection. As shown in Figure 5, after azoles treatment, the group infected with the control parental strain showed an improvement in survival compared with the untreated-azoles group (survival rate from 13% up to 80% for POS, *p* = 0.0002; from 26% up to 60% for ITC, *p* = 0.0056; from 40% up to 93% for VRC, *p* < 0.0001), indicating that using the selected azoles therapeutic dose, azoles therapy in our fungal-infection *Galleria mellonella* model was successful. In contrast, with similar therapeutic dose, POS and ITC treatment fail to increase the survival of the larvae infected with mutant strain PY01 (*p* = 0.8676 between groups without and with POS; *p* = 0.3264 between groups without and with ITC) or PY02 (*p* = 0.9067 between groups without and with POS; *p* = 0.58 between groups without and with ITC). On the other hand, after VRC treatment, the group infected with strain PY01 showed an similar improvement in survival compared with the control parental strain group (*p* < 0.0001 between PY01-infected groups without and with VRC; *p* = 0.0958 between VRC-treated PY01-infected groups and VRC-treated group infected with the control parental strain). Although the survival rate (33%) of group infected with the PY02 strain was from 13% up to 33% after VRC administration (*p* < 0.0001), it was significantly lower than survival rate (93%) of the group infected with the control parental wild-type strain after VRC administration (*p* = 0.0125). These results suggest that strains PY01 were not easily treatable with POS and ITC, and PY02 were not easily treatable with POS and ITC and VRC. These data support a conclusion that the point mutations T815A/T1378C (N248K/V436A) and T1368A (Y433N) in the *A. fumigatus cyp51A* gene are able to reduce *in vivo* the susceptibilities to azoles and decrease the efficacy of azole therapy.

## Discussion

It has found that there are varied azole drug resistance mechanisms in *A. fumigatus* including *cyp51A* mutation, overexpression of drug efflux, and modification of intracellular signaling pathways involved in stress response pathways (Wei et al., 2015). Most azole-resistant *A. fumigatus* strains are related to amino acid residue substitutions in the azole target enzyme gene *cyp51A* (Spiess et al., 2014). In this study, we indicated that *in vitro* azole situation results in emergence of azole-resistant mutations and identified unexplored *cyp51A* mutations conferring azole resistance in *A. fumigatus*.

In this study, as shown in Table 1, 94% (17/18) ITC-resistant isolates occurred *cyp51A* mutations, suggesting that *cyp51A* mutation is still main mechanism of azole resistance emergence, which is consistent with previous study. In the screening experiment for azole resistance mutant of this study, most (15/18, 83%) of drug resistant progenies generated G54E/W and M220I/K/R, which is consistent with that the G54 and M220 amino acid substitutions in the Cyp51A protein were reported as hot spot mutations (Wei et al., 2015). Different from previous reports, two undescribed mutations N248K/V436A (T815A/T1378C) and Y433N (T1368A) conferring azole resistance *in A. fumigatus* Cyp51A were detected by gene sequencing, which is not reported in previous *A. fumigatus* isolates. Previous work has showed that the V452A mutation in *C. albicans* ERG11 (equivalent of V436A mutation in *A. fumigatus* Cyp51A) does not confer *C. albicans* azole resistance (Chau et al., 2004), and the F449 mutation in *C. albicans* ERG11 (similar to the Y433 mutation in *A. fumigatus* Cyp51A based on amino-acid sequences alignment) resulted in meaningful changes in the fluconazole resistance but not in the itraconazole and voriconazole resistance (Flowers et al., 2015). Although F449 amino acid in *C. albicans* ERG11 corresponds to Y433 amino acid in *A. fumigatus* Cyp51A by amino-acid sequences blast, they are two different amino acids. In addition, the F449 amino acid site itself is not conserved in other species. Thus, we are not sure whether F449 mutation in *C. albicans* ERG11 could result in equivalent function to the Y433 mutation in *A. fumigatus* during the process of azole binding with Cyp51A. Moreover, our data from antifungal susceptibility testing for two constructed strains PY01 and PY02 by introducing point mutations into the wild-type azole-susceptible *A. fumigatus* strain *in vitro* and from *in vivo* azole treatment outcome of fungal infections in *Galleria mellonella* firstly verified that either *cyp51A^*N*248*K/V*436*A*^* or *cyp51A^*Y*433*N*^* was able to result in obviously reduce susceptibility to azoles in *A. fumigatus*.

The strain 3-339 with V436A mutation in combination with N248K and the constructed strain PY01 (*cyp51A^*N*248*K/V*436*A*^*) show high-level resistance to ITC in above *in vitro* and *in vivo* experiments. However, it is reported that N248K had been found in many azole-susceptible *A. fumigatus* strains (Liu et al., 2015) and the data from Figures 3B,D based on predicted Cyp51A protein structural model suggest that it would be difficult to deduce azole resistance with the mutation V436A or N248K/V436A. Thus, we could not confirm whether V436A mutation needs N248K mutation to confer azole resistance.

The long-term azole monotherapy is currently a primary strategy for patients with invasive aspergillosis. It is reported that clinical long-term azole therapy tends to promote the emergence of the azole-resistant strains. Whether using higher concentrations of azoles is one route to consider? Our findings indicate that under *in vitro* azole exposure, azole-resistant isolates could be recovered. In our screening experiment in this study, three original azole-susceptible *A. fumigatus* isolates were exposed to *in vitro* high-dose azole environment, and finally a few high-level azole-resistant mutants still survived. Generally, there are two possibilities for emergence of azole drug resistance. One possibility is that these resistance mutations may have originally existed in the *A. fumigatus* strains and the azole environment appears to be a selective factor for resistance mutations. The second possibility is that evolved lineages may acquire azole resistance independently during exposure of azoles (Verweij et al., 2016). Further studies are warranted to clearly verify whether azole environment just play a selective role or other unknown functions for resistance mutations. In our screening experiment, the final 18 isolates exhibited different and independent colony phenotypes compared to the reference strain. Among them, 17 isolates had mutations in cyp51A but one strain retained the wild-type cyp51A gene (strain 1160-132). In our further future study, we may check whether exist the non-*cyp51A*-mediated azole resistance mechanism in strain 1160-132.

## Conclusion

In conclusion, our data in this study indicated that *in vitro* azole situation results in emergence of azole-resistant mutations and there are unexplored *cyp51A* mutants conferring azole resistance in *A. fumigatus*.

## Data Availability Statement

The datasets generated for this study can be found in the GenBank—accession numbers for sequences of ITS, β-tubulin, and calmodulin of clinical isolates NO.3 is respectively MH536092, MH536090, and MH536094. Those for NO.7 is respectively MH536093, MH536091, and MH536095.

## Ethics Statement

This study was carried out in accordance with the recommendations of the Guide for the Care and Use of Laboratory Animals of the National Institutes of Health, United States. The protocol was reviewed and approved by the Animal Care and Use Program at Jinling Hospital, China (protocol number 2018GKJDWLS-03-024).

## Author Contributions

PC, QZ, ZZ, WL, and MS completed the experiments. PC, HS, and LL conducted the experiments and data analysis. PC and LL wrote the manuscript. All authors read and approved the manuscript.

## Conflict of Interest

The authors declare that the research was conducted in the absence of any commercial or financial relationships that could be construed as a potential conflict of interest.

## Footnotes

1 http://fungi.ensembl.org/

## References

[B1] Arendrup M. C. (2014). Update on antifungal resistance in *Aspergillus* and *Candida*. *Clin. Microbiol. Infect.* 20(Suppl. 6), 42–48. 10.1111/1469-0691.12513 24372701

[B2] Becher R. Wirsel S. G. (2012). Fungal cytochrome P450 sterol 14alpha-demethylase (CYP51) and azole resistance in plant and human pathogens. *Appl. Microbiol. Biotechnol.* 95 825–840. 10.1007/s00253-012-4195-9 22684327

[B3] Bowyer P. Moore C. B. Rautemaa R. Denning D. W. Richardson M. D. (2011). Azole antifungal resistance today: focus on *Aspergillus*. *Curr. Infect. Dis. Rep.* 13 485–491. 10.1007/s11908-011-0218-4 21931980

[B4] Camps S. M. Van Der Linden J. W. Li Y. Kuijper E. J. Van Dissel J. T. Verweij P. E. (2012). Rapid induction of multiple resistance mechanisms in *Aspergillus fumigatus* during azole therapy: a case study and review of the literature. *Antimicrob. Agents Chemother.* 56 10–16. 10.1128/AAC.05088-11 22005994 PMC3256077

[B5] Chau A. S. Mendrick C. A. Sabatelli F. J. Loebenberg D. Mcnicholas P. M. (2004). Application of real-time quantitative PCR to molecular analysis of *Candida albicans* strains exhibiting reduced susceptibility to azoles. *Antimicrob. Agents Chemother.* 48 2124–2131. 10.1128/aac.48.6.2124-2131.2004 15155210 PMC415610

[B6] Chen J. Li H. Li R. Bu D. Wan Z. (2005). Mutations in the cyp51A gene and susceptibility to itraconazole in *Aspergillus fumigatus* serially isolated from a patient with lung aspergilloma. *J. Antimicrob. Chemother.* 55 31–37. 10.1093/jac/dkh507 15563516

[B7] Chowdhary A. Sharma C. Hagen F. Meis J. F. (2014). Exploring azole antifungal drug resistance in *Aspergillus fumigatus* with special reference to resistance mechanisms. *Future Microbiol.* 9 697–711. 10.2217/fmb.14.27 24957095

[B8] Chowdhary A. Sharma C. Meis J. F. (2017). Azole-Resistant Aspergillosis: epidemiology. Molecular mechanisms, and treatment. *J. Infect. Dis.* 216 S436–S444. 10.1093/infdis/jix210 28911045

[B9] Clinical and Laboratory Standards Institute (2008). *Reference Method for Broth Dilution Antifungal Susceptibility Testing of Filamentous Fungi; Approved Standard*, 2nd Edn Wayne, PA: Clinical and Laboratory Standards Institute.

[B10] Cowen L. E. Sanglard D. Howard S. J. Rogers P. D. Perlin D. S. (2015). Mechanisms of antifungal drug resistance. *Cold Spring Harb. Perspect. Med.* 5:a019752. 10.1101/cshperspect.a019752 25384768 PMC4484955

[B11] Flowers S. A. Colon B. Whaley S. G. Schuler M. A. Rogers P. D. (2015). Contribution of clinically derived mutations in ERG11 to azole resistance in *Candida albicans*. *Antimicrob. Agents Chemother.* 59 450–460. 10.1128/AAC.03470-14 25385095 PMC4291385

[B12] Garcia-Rubio R. Cuenca-Estrella M. Mellado E. (2017). Triazole Resistance in *Aspergillu*s Species: an emerging problem. *Drugs* 77 599–613. 10.1007/s40265-017-0714-4 28236169

[B13] Godet C. Laurent F. Bergeron A. Ingrand P. Beigelman-Aubry C. Camara B. (2016). CT imaging assessment of response to treatment in chronic pulmonary aspergillosis. *Chest* 150 139–147. 10.1016/j.chest.2016.02.640 26905365

[B14] Hagiwara D. Watanabe A. Kamei K. Goldman G. H. (2016). Epidemiological and genomic landscape of azole resistance mechanisms in *Aspergillus* Fungi. *Front. Microbiol.* 7:1382. 10.3389/fmicb.2016.01382 27708619 PMC5030247

[B15] Howard S. J. Cerar D. Anderson M. J. Albarrag A. Fisher M. C. Pasqualotto A. C. (2009). Frequency and evolution of Azole resistance in *Aspergillus fumigatus* associated with treatment failure. *Emerg. Infect. Dis.* 15 1068–1076. 10.3201/eid1507.090043 19624922 PMC2744247

[B16] Jiang H. Shen Y. Liu W. Lu L. (2014). Deletion of the putative stretch-activated ion channel *Mid1* is hypervirulent in *Aspergillus fumigatus*. *Fungal Genet. Biol.* 62 62–70. 10.1016/j.fgb.2013.11.003 24239700

[B17] Krishnan Natesan S. Wu W. Cutright J. L. Chandrasekar P. H. (2012). In vitro–in vivo correlation of voriconazole resistance due to G448S mutation (*cyp51A* gene) in *Aspergillus fumigatus*. *Diagn. Microbiol. Infect. Dis.* 74 272–277. 10.1016/j.diagmicrobio.2012.06.030 22897872

[B18] Liu M. Zeng R. Zhang L. Li D. Lv G. Shen Y. (2015). Multiple *cyp51A*-based mechanisms identified in azole-resistant isolates of *Aspergillus fumigatus* from China. *Antimicrob. Agents Chemother.* 59 4321–4325. 10.1128/AAC.00003-15 25896700 PMC4468671

[B19] Liu M. Zheng N. Li D. Zheng H. Zhang L. Ge H. (2016). *cyp51A*-based mechanism of azole resistance in *Aspergillus fumigatus*: Illustration by a new 3D Structural Model of *Aspergillus fumigatus* CYP51A protein. *Med. Mycol.* 54 400–408. 10.1093/mmy/myv102 26768370

[B20] Mann P. A. Parmegiani R. M. Wei S. Q. Mendrick C. A. Li X. Loebenberg D. (2003). Mutations in *Aspergillus fumigatus* resulting in reduced susceptibility to posaconazole appear to be restricted to a single amino acid in the cytochrome P450 14alpha-demethylase. *Antimicrob. Agents Chemother.* 47 577–581. 10.1128/aac.47.2.577-581.2003 12543662 PMC151774

[B21] Moye-Rowley W. S. (2015). Multiple mechanisms contribute to the development of clinically significant azole resistance in *Aspergillus fumigatus*. *Front. Microbiol.* 6:70. 10.3389/fmicb.2015.00070 25713565 PMC4322724

[B22] Shao P. L. Huang L. M. Hsueh P. R. (2007). Recent advances and challenges in the treatment of invasive fungal infections. *Int. J. Antimicrob. Agents* 30 487–495. 10.1016/j.ijantimicag.2007.07.019 17961990

[B23] Sharma C. Hagen F. Moroti R. Meis J. F. Chowdhary A. (2015). Triazole-resistant *Aspergillus fumigatus* harbouring G54 mutation: is it de novo or environmentally acquired? *J. Glob. Antimicrob. Resist.* 3 69–74. 10.1016/j.jgar.2015.01.005 27873672

[B24] Spiess B. Postina P. Reinwald M. Cornely O. A. Hamprecht A. Hoenigl M. (2014). Incidence of *Cyp51* A key mutations in *Aspergillus fumigatus*-a study on primary clinical samples of immunocompromised patients in the period of 1995-2013. *PLoS One* 9:e103113. 10.1371/journal.pone.0103113 25072733 PMC4114486

[B25] Tashiro M. Izumikawa K. Hirano K. Ide S. Mihara T. Hosogaya N. (2012). Correlation between triazole treatment history and susceptibility in clinically isolated *Aspergillus fumigatus*. *Antimicrob. Agents Chemother.* 56 4870–4875. 10.1128/AAC.00514-12 22751542 PMC3421857

[B26] Vazquez J. A. Manavathu E. K. (2016). Molecular characterization of a voriconazole-resistant, posaconazole-susceptible *Aspergillus fumigatus* Isolate in a lung transplant recipient in the United States. *Antimicrob. Agents Chemother.* 60 1129–1133. 10.1128/AAC.01130-15 26574014 PMC4750678

[B27] Verweij P. E. Zhang J. Debets A. J. M. Meis J. F. Van De Veerdonk F. L. Schoustra S. E. (2016). In-host adaptation and acquired triazole resistance in *Aspergillus fumigatus*: a dilemma for clinical management. *Lancet Infect. Dis.* 16 e251–e260. 10.1016/S1473-3099(16)30138-4 27638360

[B28] Walsh T. J. Anaissie E. J. Denning D. W. Herbrecht R. Kontoyiannis D. P. Marr K. A. (2008). Treatment of aspergillosis: clinical practice guidelines of the Infectious Diseases Society of America. *Clin. Infect. Dis.* 46 327–360. 10.1086/525258 18177225

[B29] Wei X. Zhang Y. Lu L. (2015). The molecular mechanism of azole resistance in *Aspergillus fumigatus*: from bedside to bench and back. *J. Microbiol.* 53 91–99. 10.1007/s12275-015-5014-7 25626363

[B30] Wei X. Chen P. Gao R. Li Y. Zhang A. Liu F. (2017). Screening and Characterization of a Non-cyp51A Mutation in an *Aspergillus fumigatus* cox10 Strain Conferring Azole Resistance. *Antimicrob. Agents Chemother.* 61:e02101-16. 10.1128/AAC.02101-16 27799210 PMC5192120

