# Supplementary material for: Uncovering New Mutations Conferring Azole Resistance in the Aspergillus fumigatus cyp51A Gene
Source: Front Microbiol. 2020 Jan 21;10:3127. doi: 10.3389/fmicb.2019.03127 (PMC6986205; doi:10.3389/fmicb.2019.03127)
Supplement: Supplementary file 1 [file Data_Sheet_1.pdf]

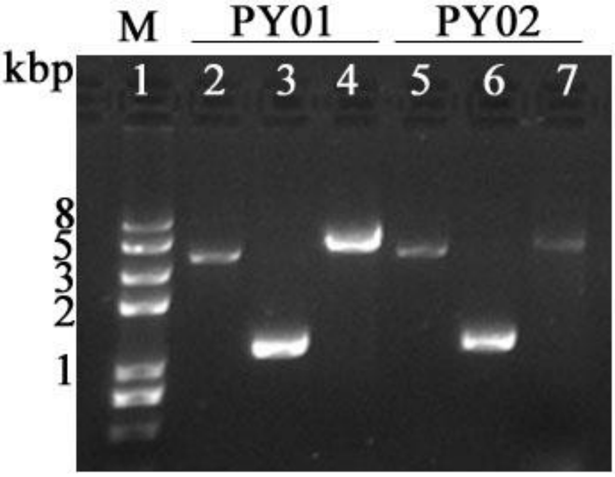

Figure S1 PCR analysis showing the integration of the pyrG::*cyp51A*$^{point\ mutation}$ fragment into the genome at the original *cyp51A* locus in the PY01 or PY02 strains.

For lanes 2 and 5, the primers cyp51A-F1 and Diag-Ncpyr4-3’ were used to determine whether there was a homologous recombination to replace *cyp51A* with pyrG::*cyp51A*$^{point\ mutation}$ fragment in the genome of PY01 or PY02, and the expected size is 3686 bp; For lanes 3 and 6, primers Diag-Ncpyr4-5’ and P6 were used to determine whether there was a homologous recombination to replace *cyp51A* with pyrG::*cyp51A*$^{point\ mutation}$ fragment in the genome of PY01 or PY02, and the expected size is 1133 bp; For lanes 4 and 7, primers cyp51A-F and cyp51A-R2 were used to determine whether wild-type gene *cyp51A* still exists in the genome of PY01 or PY02, and the expected size of wild-type gene *cyp51A* is 2113 bp, and the expected size of pyrG::*cyp51A*$^{point\ mutation}$ is 4321 bp. In lanes 2-4, genomic DNA of PY01 was used as a PCR template; for lanes 5-7, genomic DNA of PY02 was used as a PCR template.

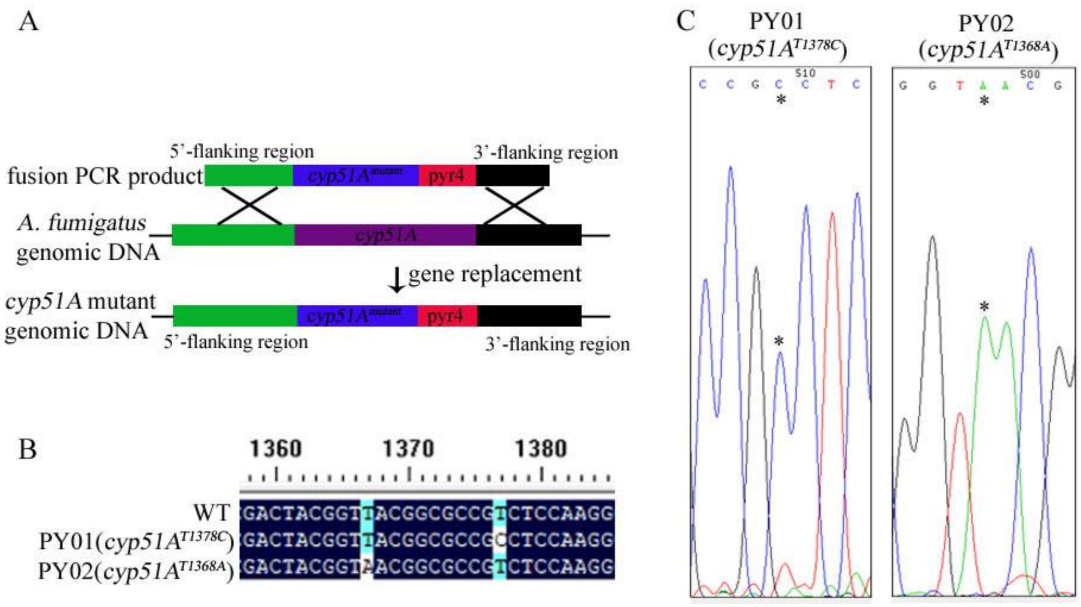

Figure S2 The construction of point mutations PY01 (*cyp51A*$^{N248K/V436A}$) and PY02 (*cyp51A*^{Y433N}) and sequencing analysis for *cyp51A* gene. (A) Diagram showing the gene replacement strategy for mutations. (B, C) The *cyp51A* gene sequencing analysis showing expected mutation sites in the PY01 and PY02 strains. Asterisk (C) show mutation sites.

TABLE S1 Primers used in this study

Primer name	DNA sequence 5'-3'
cyp51A-F1	CTCAGGACTTTCTTCTAACCACCAT
cyp51A-R1	TACAGTCATTTATTAGGCCCTCGAG
cyp51A-F2	GCAGCACCACTTCAGAGTTGTCTAG
cyp51A-R2	AGTATAGGCAACAACACTTCAGGGC
P2	AAGACTTGGCTTAGCTCCCTTACCG
P3	GTAACGCCAGGGTTTTCCCAGTCTCACTTGGATGTGTTTTTCG
P4	TGCTCTGATGCCGCATAGTTGTAACCATCGAGGACTTCAAAGG
P5	CCGTCCACGTATGACATGCATATG
P6	TCTCGTGTGACTATGACCCCGT
Pyr4-up	GGAAAACCCTGGCGTTAC
Pyr4-down	CTATGCGGCATCAGAGCA
Diag-Ncpyr4-5'	GTTGCATTCGATAAGTAGCCAGTTC
Diag-Ncpyr4-3'	GTCTTGAGGACGACAATCGA

TABLE S2 *A. fumigatus* strains used in this study

Strain	Genotype	Reference or source
ATCC MYA-3626	NA	ATCC
NO.3	NA	This study
NO.7	NA	This study
A1160[c]	*Δku80; A1160::pyrG; veA1*	(Jiang et al., 2014)
A1160	*Δku80; pyrG; veA1*	FGSC
PY01	*Δku80; pyrG; cyp51A*$^{N248K/V436A}$*::pyr4; veA1*	This study
PY02	*Δku80; pyrG; cyp51A*^{Y433N}*::pyr4; veA1*	This study

FGSC: Fungal Genetics Stock Center (http://www.fgsc.net/); NA: not applicable; ATCC: American Type Culture Collection.
